# Vick’s Floral Guide

**Published:** 1879-01-20

**Authors:** 


### *VICK'S FLORAL GUIDE.*

This annual visitant again greets us with its cheerful and beautifully illustrated pages. A neat little work of 100 pages, a large number of attractive illustrations and one very beautiful colored flower plate.

There is a full description of all the flowers and vegetables known in this country. A five cent stamp will secure this neat little work. Address Jas. Vick, Rochester, N. Y.

